# A bibliometric analysis of research hotspots and trends in transcranial magnetic stimulation and Alzheimer’s disease

**DOI:** 10.3389/fnagi.2025.1544702

**Published:** 2025-02-27

**Authors:** Dingwen Xu, Yang Feng, Zhihua Lu, Ruijia Ma, Weicai Zhang, Zhen Mou, Lingling Zhang, Xiufeng Tang, Zhenxiong Zhao, Zhencang Zheng

**Affiliations:** ^1^Department of Clinic, School of Medicine, Yangzhou Polytechnic College, Yangzhou, China; ^2^Department of Geriatrics, The Third Hospital of Santai, Mianyang, China; ^3^Tianjin Key Laboratory of Agricultural Animal Breeding and Healthy Husbandry, College of Animal Science and Veterinary Medicine, Tianjin Agricultural University, Tianjin, China; ^4^Taizhou Central Hospital (Taizhou University Hospital), Taizhou, China; ^5^Department of Pharmacy and Shandong Provincinal Key Traditional Chinese Medical Discipline of Clinical Chinese Pharmacy, Shandong Cancer Hospital and Institute, Shandong First Medical University and Shandong Academy of Medical Sciences, Jinan, China

**Keywords:** bibliometric analysis, transcranial magnetic stimulation, Alzheimer’s disease, publication, mild cognitive impairment, dementia, memory, double-blind

## Abstract

**Background:**

Research regarding Transcranial Magnetic Stimulation (TMS) and Alzheimer’s disease (AD) has been increasing; however, no bibliometric analysis has yet been conducted in this domain. This study employs bibliometric methods to identify research trends and hotspots concerning AD and TMS.

**Methods:**

We conducted a search in the Web of Science Core Database for articles related to AD and TMS from January 1, 2014, to October 22, 2024. After stringent selection, we performed bibliometric analysis using Excel, VOSviewer, CiteSpace and CoreMine.

**Results:**

The number of articles pertaining to AD and TMS has increased annually, with a notable surge post-2020. The three leading countries in publication volume are China, the United States, and Italy. The top institutions contributing to this field include Harvard Medical School, the University of Toronto, and the University of Brescia. The author with the highest publication output is Giacomo Koch. The journal with the most publications is the Journal of Alzheimer’s disease. The 10 most frequently occurring keywords are Alzheimer’s disease, Transcranial Magnetic Stimulation, mild cognitive impairment, dementia, memory, double-blind, repetitive transcranial magnetic stimulation, noninvasive brain stimulation, cognitive impairment, and plasticity. Text mining has revealed that the anatomical structure “brain” and the gene “Amyloid Precursor Protein (APP)” are significantly related to both AD and TMS, suggesting that TMS may offer a therapeutic avenue for AD by modulating the activity of APP.

**Conclusion:**

Our article employs bibliometric methods to unveil trends in research related to AD and TMS, including collaborations among countries, regions, and authors, as well as key research hotspots. We provide objective data that serves as a reference for scientific research and clinical work concerning AD and TMS.

## 1 Introduction

Alzheimer’s disease (AD) represents a progressive neurodegenerative disorder and is the most prevalent type of dementia. The hallmark pathological features of AD are the deposition of amyloid plaques, known as senile plaques (SP), and the formation of neurofibrillary tangles (NFTs) due to hyperphosphorylated tau proteins. These lead to progressive memory decline and subsequent loss of cognitive functions (Scheltens et al., 2021). With the average human lifespan extending, the prevalence of AD has surged in recent decades. By the early 21st century, over 50 million individuals worldwide suffered from dementia, with projections suggesting this number will exceed 150 million by mid-century (Graff-Radford et al., 2021). The high incidence and disability rates of AD render it a significant public health concern, increasingly becoming a societal burden and a complex medical issue globally (Tahami Monfared et al., 2022).

Although the FDA has approved several medications for the treatment of dementia, none effectively alleviate symptoms. Current pharmacological interventions include cholinesterase inhibitors and excitatory amino acid receptor antagonists. The former comprises Donepezil, Galantamine, and Rivastigmine, while the latter mainly includes Memantine (Khan et al., 2020). Additionally, various strategies, such as vaccines, antibodies, and *γ*- or *β*-secretase inhibitors, can effectively reduce Aβ deposition in the brain. However, their failure to yield clinical benefits has resulted in numerous unsuccessful clinical trials (Mullard, 2021). Due to the lack of breakthrough advancements in pharmacological treatment, there is an urgent clinical need for safe and effective adjunctive therapies. Electroconvulsive therapy (ECT) has been shown to exhibit some efficacy for AD, but its side effects and invasive nature make it less acceptable to patients and families, and its long-term application is challenging, limiting its broader clinical use (Chang et al., 2018).

Transcranial Magnetic Stimulation (TMS) serves as an effective method for modulating brain networks, capable of altering local cortical excitability and demonstrating “remote effects,” meaning that the effects induced by TMS are not confined to the stimulated cortex; rather, distant brain regions connected to the stimulated cortex can also be concurrently regulated. For instance, a study integrating functional magnetic resonance imaging (fMRI) with TMS to evaluate visuospatial judgment revealed a notable correlation between the induced behavioral deficits and the fMRI alterations in both the directly stimulated parietal and the remote ipsilateral frontal cortical areas (Hallett, 2007). Research indicates that TMS holds potential in improving clinical symptoms of AD and slowing disease progression. Excitatory TMS sequences can enhance cognitive functions in AD patients (Chou et al., 2022). TMS stimulation targets regions associated with depression and can alleviate depressive symptoms in AD patients while inducing changes in functional connectivity (Matt et al., 2022). As an alternative to ECT, TMS is utilized in the treatment of various neuropsychiatric disorders, including AD, depression, stroke, epilepsy, and post-traumatic stress disorder (Vaishnavi, 2023). Therefore, the application of TMS in AD presents significant potential, and further investigation into this area warrants our attention. Such investigations could yield valuable insights into the mechanisms of action, optimal treatment protocols, and potential long-term benefits of TMS, thereby enhancing our understanding and therapeutic strategies for AD.

Despite the growing interest in AD and TMS, the field currently lacks bibliometric analyses that objectively assess TMS applications for AD. This limitation hampers our understanding of the development, trending topics, and future prospects of TMS in relation to AD. Therefore, this study employs bibliometric methods for the first time to analyze research trends within the domains of AD and TMS. It aims to provide scholars with insights into advancements and breakthroughs in the field and guide further investigations on applying TMS in the treatment of AD.

## 2 Methods

### 2.1 Literature search and selection strategy

**Figure 1:**
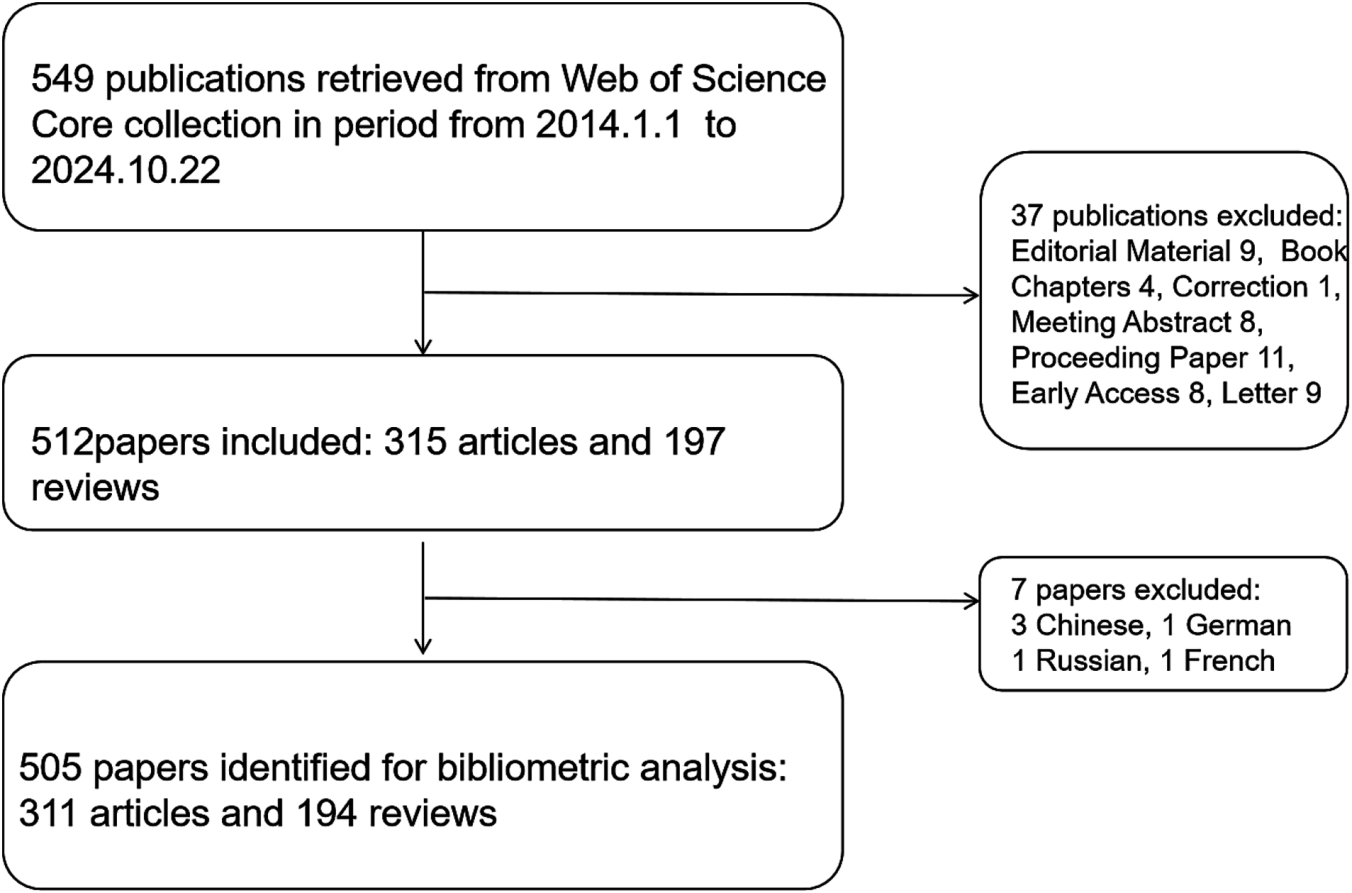
Schematic diagram of the literature search and selection methodology.

We searched the Web of Science Core Collection database, covering the period from January 1, 2014, to October 22, 2024. The search terms were TS = (Alzheimer’s disease) or (Alzheimer Disease) OR (Alzheimer Dementias) OR (Senile Dementia) AND TS = (Transcranial Magnetic Stimulations) OR (Magnetic Stimulations, Transcranial) OR (Magnetic Stimulation, Transcranial) OR (Stimulations, Transcranial Magnetic) OR (Stimulation, Transcranial Magnetic) OR (Transcranial Magnetic Stimulation, Paired Pulse) OR (Transcranial Magnetic Stimulation, Repetitive) OR (Transcranial Magnetic Stimulation, Single Pulse). Inclusion criteria limited publication types to “Article” and “Review”; language restricted to English. Exclusion criteria encompassed Editorial Material, Book Chapters, Correction, Meeting Abstract, Proceeding Paper, Early Access, and Letter. After screening, we selected “Export Records to Plain Text File.” Two authors independently conducted literature screening and data extraction to minimize subjective bias. For any disputed papers, discussions with a third author were held to reach consensus (Figure 1).

### 2.2 Bibliometric analysis

We employed GraphPad Prism 10, an online bibliometric analysis platform,^1^ VOSviewer (v1.6.19), and CiteSpace (v.6.1.R6) for bibliometric and visualization analysis. Yearly publication volume was statistically analyzed using GraphPad Prism 10, while the online bibliometric platform provided insights into inter-country collaboration and annual publication statistics for high-output nations. VOSviewer (v1.6.19) facilitated collaborative network analysis among countries, institutions, and authors. CiteSpace (v6.1.R6) was utilized for clustering and citation burst analysis.

### 2.3 Text mining analysis

The text mining utilized the CoreMine platform, which is based on existing literature. The indexed text records consist of those that form the MEDLINE database, specifically the titles and abstracts included in PubMed. We employed the online tool CoreMine^2^ to mine relevant literature, analyzing items significantly related to both AD and TMS. In the output figure, the “Filter by connection relevance” criterion is set at a *p*-value of less than 0.05, with thicker lines representing higher relevance, indicating a closer association between the two terms.

## 3 Results

### 3.1 Analysis of annual publications

**Figure 2:**
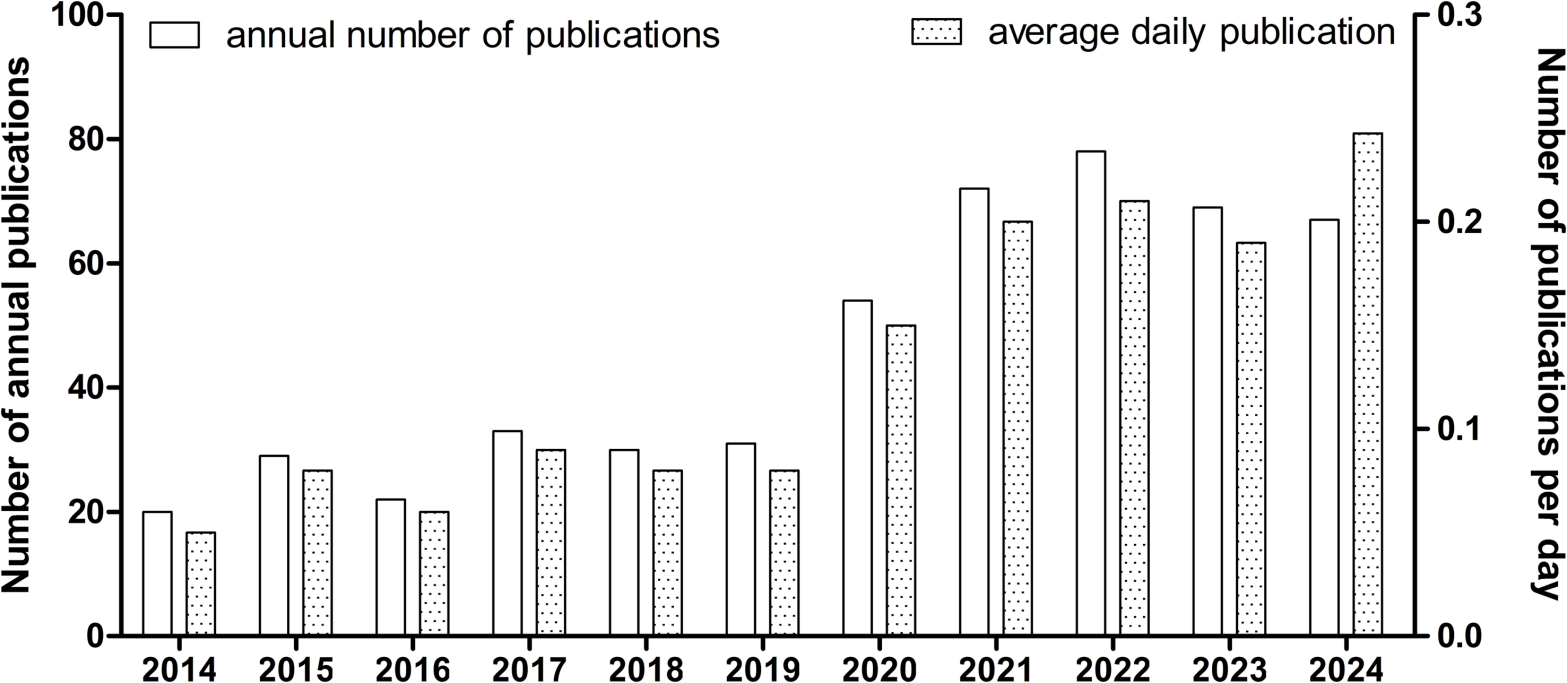
Evaluation of yearly scholarly outputs.

From January 1, 2014, to October 22, 2024, a total of 505 articles meeting our selection criteria were published, including 311 articles and 194 reviews, as shown in Figure 1. Before 2020, the number of publications each year remained below 50, experiencing slow growth. However, post-2020, the publication rate surged dramatically, reaching 78 articles in 2022—more than double that of 2019. There was a slight decrease in 2023, but by the time of this research, 67 articles had already been published in 2024. The average daily publication rate provides a clearer picture of the publication trend for 2024. We observed that in 2014, the average was 0.05 articles per day. This increased to 0.21 articles per day in 2022 and slightly decreased to 0.19 in 2023, with 2024 averaging 0.24 articles per day (Figure 2). Therefore, despite occasional minor fluctuations, the overall number of articles regarding AD and TMS shows a consistent annual increase, especially notable after 2020.

### 3.2 Analysis of countries

**Figure 3:**
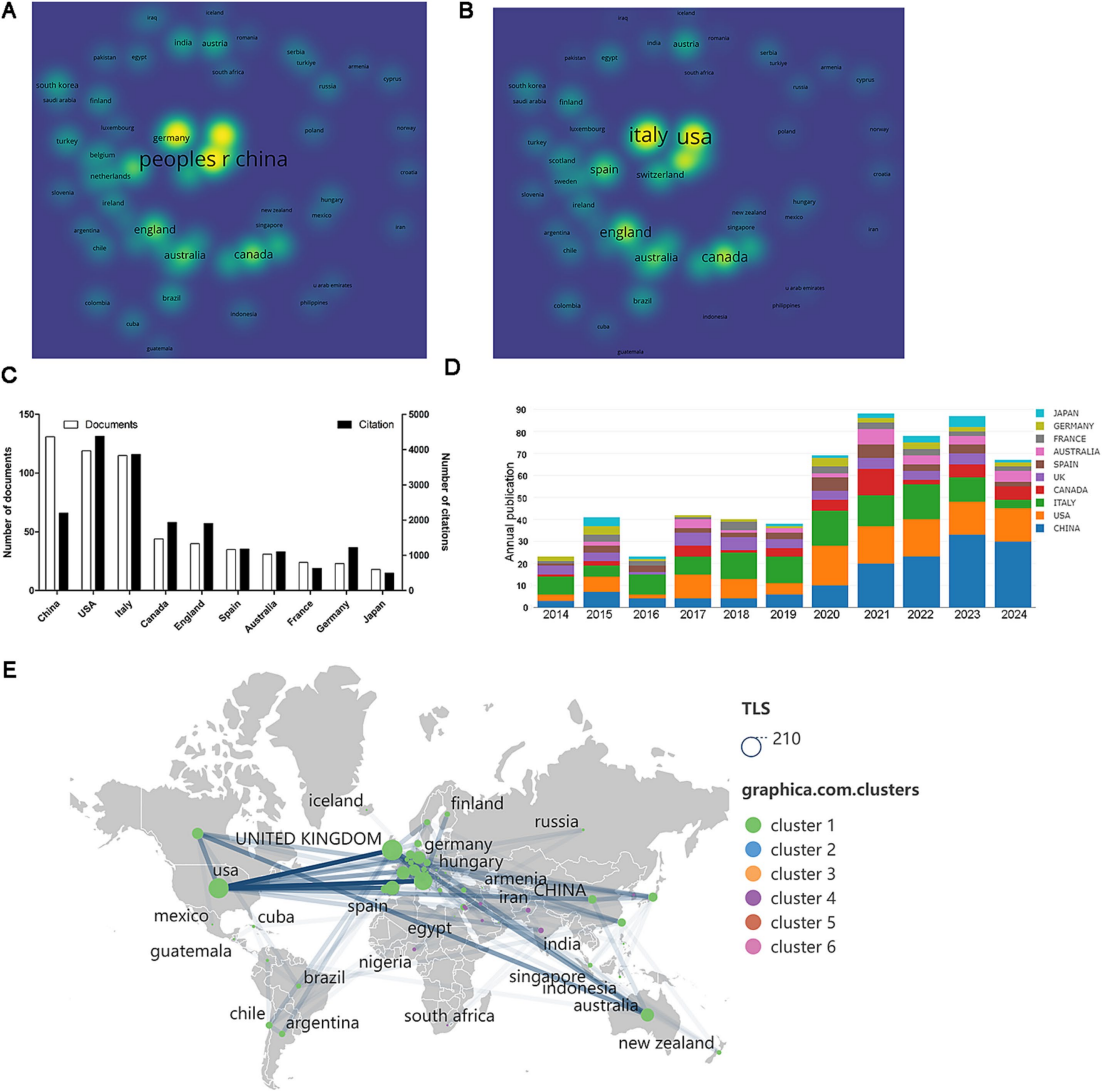
Analysis of countries. **(A)** Visual representations of density based on the number of documents. **(B)** Visual representations of density based on citation counts. **(C)** The 10 leading countries ranked by publication volume. **(D)** Yearly publication for the top 10 countries. **(E)** Co-authorship analysis illustrated in the geographical map.

A total of 61 countries/regions contributed to this field. The number of publications and their citation counts can be illustrated using density visualization (Figures 3A,B). As shown in Figure 3C, the top 10 countries by publication count are led by China with 131 papers, followed by the United States (*n* = 119) and Italy (*n* = 115). In terms of total citations, the leading three countries are the United States (*n* = 4,378), Italy (*n* = 3,863), and Canada (*n* = 1935). Furthermore, the annual publication output of these 10 countries has increased year by year, with China showing the most significant annual growth (Figure 3D). Co-authorship analysis of these nations identifies the three countries with the highest collaboration levels as the United States (TLS = 181), Italy (TLS = 152), and the United Kingdom (TLS = 121). Among these, the United States has the most robust collaborative network with European countries or regions (Figure 3E).

### 3.3 Analysis of organizations

**Figure 4:**
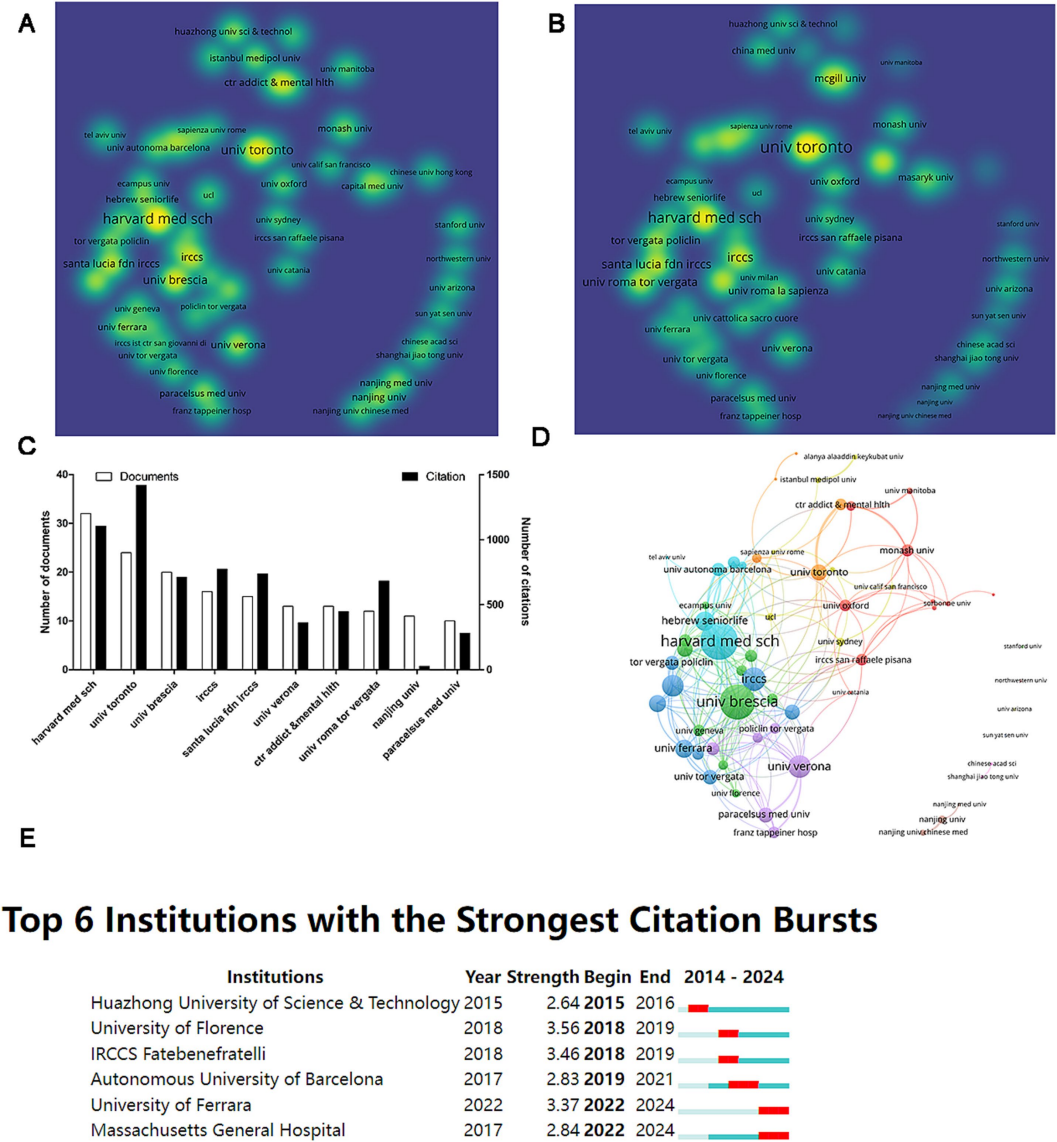
Analysis of academic institutions. **(A)** Visualization map of density based on document counts. **(B)** Visualization map of density based on citation counts. **(C)** Ranking of the top 10 institutions by publication output. **(D)** Network visualization of institutional collaborations. **(E)** Citation burst analysis of institutions.

A total of 1,006 institutions published articles on AD and TMS. Among these, 61 institutions contributed more than five papers. A co-authorship analysis visualizes these 61 institutions, creating density visualization diagrams based on publication and citation counts (Figures 4A,B). The top 10 institutions ranked by publication volume include Harvard Medical School, which has the highest output with 32 articles, followed by the University of Toronto (*n* = 24) and the University of Brescia (*n* = 20). The citation count shows the University of Toronto leads with 1,417 citations (Figure 4C). Subsequently, the network visualization reveals that these high-output institutions maintain a close collaborative network, particularly Harvard Medical School (TLS = 46) and the University of Brescia (TLS = 41), indicating strong inter-institutional relationships. In contrast, Stanford University, Northwestern University, the University of Arizona, and Sun Yat-sen University exhibit no collaborative ties with other institutions (Figure 4D). The Citation Bursts analysis identifies six institutions experiencing citation bursts, with the University of Florence demonstrating the strongest burst (strength = 3.56). The University of Ferrara and Massachusetts General Hospital are currently in a citation burst phase.

### 3.4 Analysis of authors

**Figure 5:**
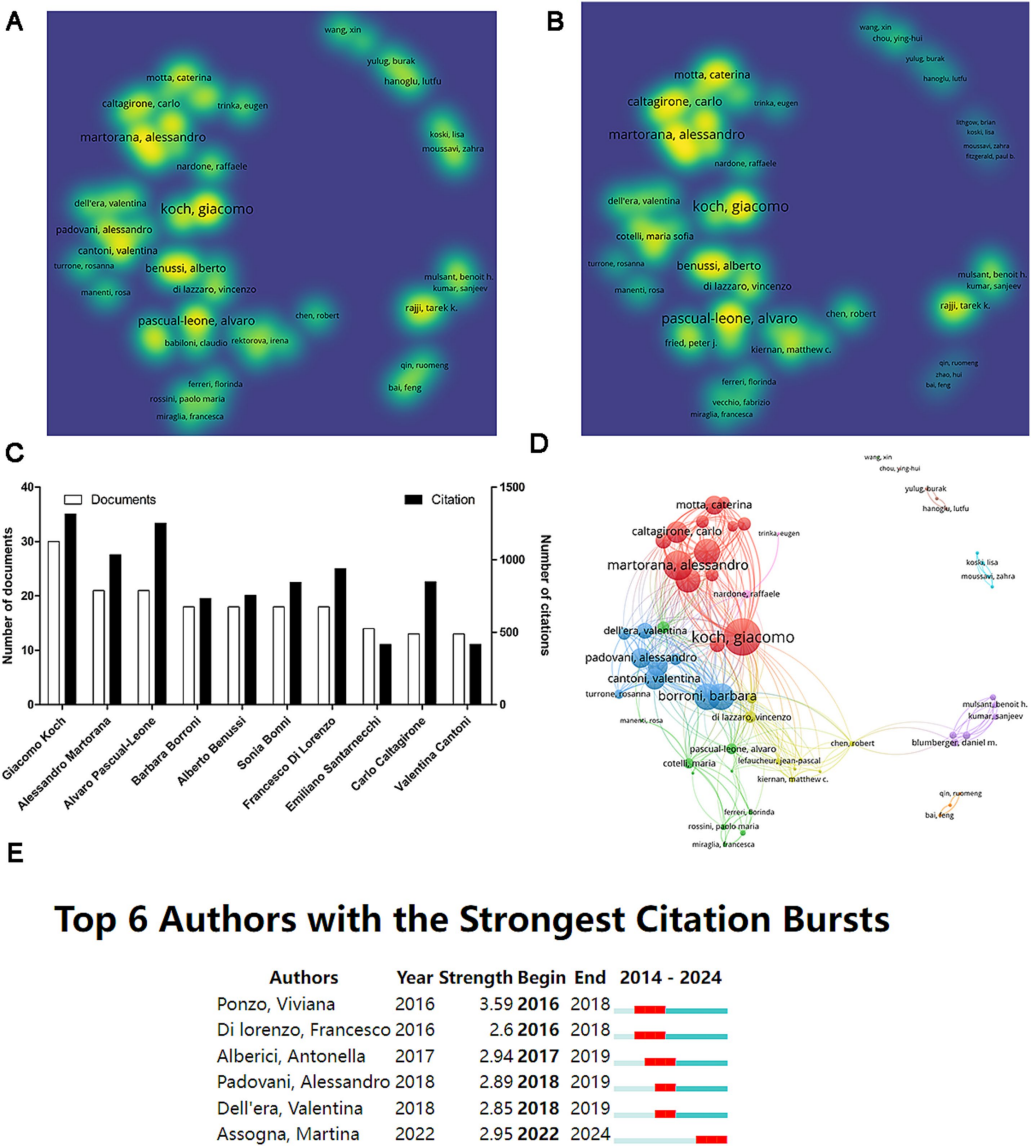
Analysis of authors. **(A)** Density visualization map based on document counts. **(B)** Density visualization map based on citation counts. **(C)** The top 10 authors ranked by publication volume. **(D)** Network visualization illustrating author collaborations. **(E)** Citation burst analysis of author.

A total of 2,684 authors contributed articles on AD and TMS, with 60 authors publishing more than five papers. Conducting a co-authorship analysis on these 60 authors, their output and citation metrics are presented using density visualizations (Figures 5A,B). The top 10 authors ranked by output include Giacomo Koch from the Santa Lucia Foundation IRCCS, who leads with 30 papers and the highest citations (*n* = 1,315). He is followed by Alessandro Martorana from the University of Rome “Tor Vergata” and Alvaro Pascual-Leone from Harvard Medical School, both having published 21 papers (Figure 5C). Using network visualization, we illustrate the collaboration among these prolific authors. Generally, authors with high publication counts also show close collaboration with others; for example, Giacomo Koch, having the highest publication volume, maintains the strongest collaborative ties (TLS = 186) (Figure 5D). Citation burst analysis reveals that six authors experienced citation bursts, with Viviana Ponzo exhibiting the strongest burst (strength = 3.59), while Martina Assogna is currently in a citation burst period (Figure 5E).

### 3.5 Analysis of journals

**Figure 6:**
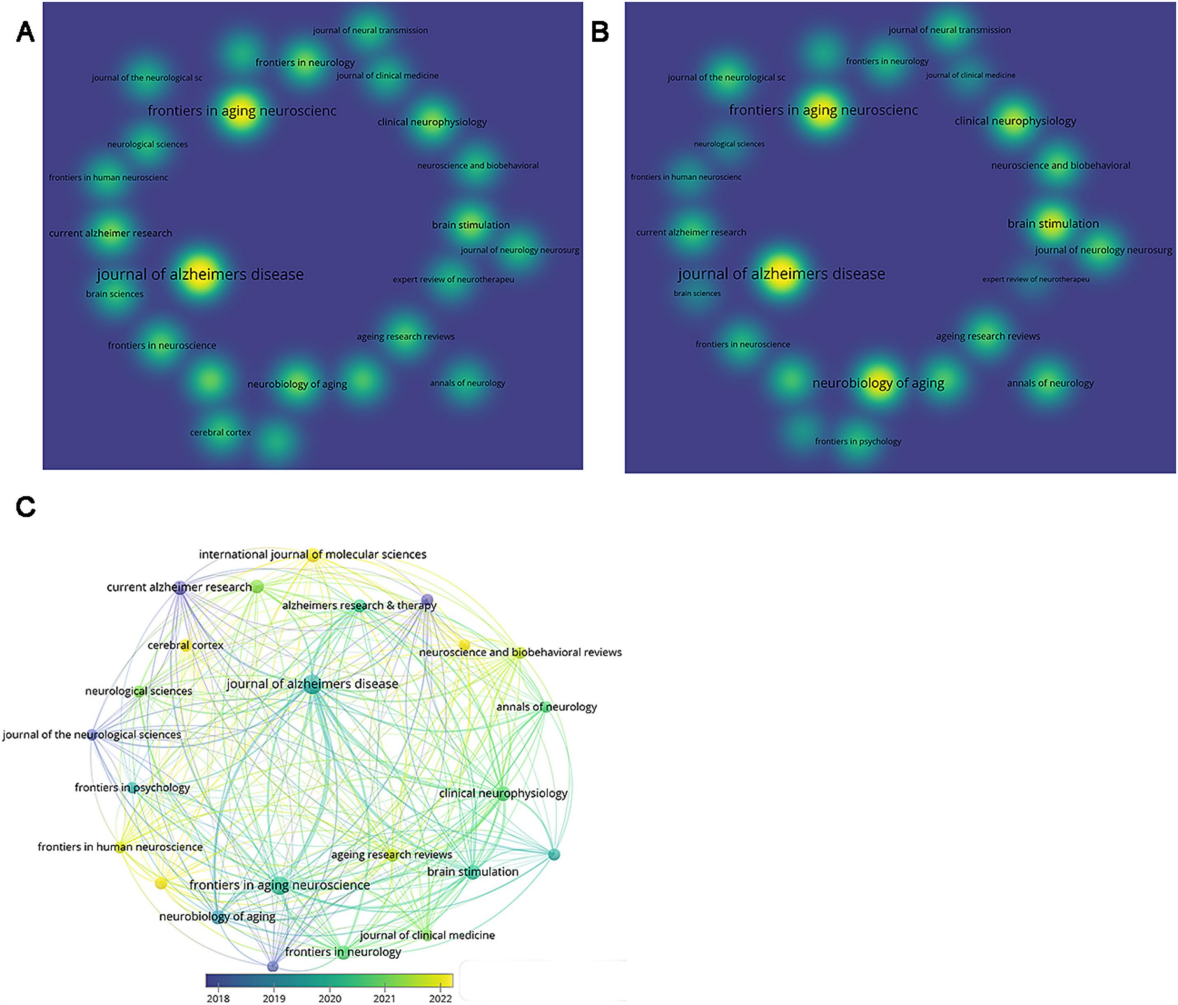
Analysis of journals. **(A)** Density visualization map of journals based on document counts. **(B)** Density visualization map of journals based on citation counts. **(C)** The overlay visualization of bibliographic coupling between academic journals.

A total of 219 journals contributed articles on AD and TMS, with 24 journals publishing more than five articles. Their publication and citation volumes are illustrated in the density visualization (Figures 6A,B). The Journal of Alzheimer’s disease had the highest publication count, totaling 36 articles, followed by Frontiers in Aging Neuroscience with 30 articles, and Clinical Neurophysiology with 12 articles. The citation counts for these three journals were 730, 724, and 402, respectively. The top 10 journals in terms of publication volume are shown in Table 1, with the highest impact factor being 12.5 for Ageing Research Reviews. Among the journals, four belong to the Q1 category, five to Q2, and one to Q3 in JCR ranking. VOSviewer performed a bibliographic coupling analysis on journals with more than five publications and found that the Journal of the Neurological Sciences, Current Alzheimer Research, and Journal of Neurology were prominent in early publication counts. In contrast, Brain Sciences, International Journal of Molecular Sciences, and Cerebral Cortex showed higher publication counts recently (Figure 6C).

**Table 1:** Top 10 journals by publication output.

Source	Documents	Citations	Impact factor (2023)	JCR
Journal of Alzheimer’s disease	36	930	3.4	Q2
Frontiers in aging neuroscience	30	724	4.1	Q2
Clinical neurophysiology	12	402	3.7	Q1
Brain stimulation	12	500	7.6	Q1
Frontiers in neurology	11	139	2.7	Q2
Neurobiology of aging	11	655	3.7	Q2
Current Alzheimer’s research	11	188	1.8	Q3
International journal of molecular sciences	10	198	4.9	Q1
Frontiers in neuroscience	9	151	3.2	Q2
Ageing research reviews	8	225	12.5	Q1

### 3.6 Analysis of references

**Figure 7:**
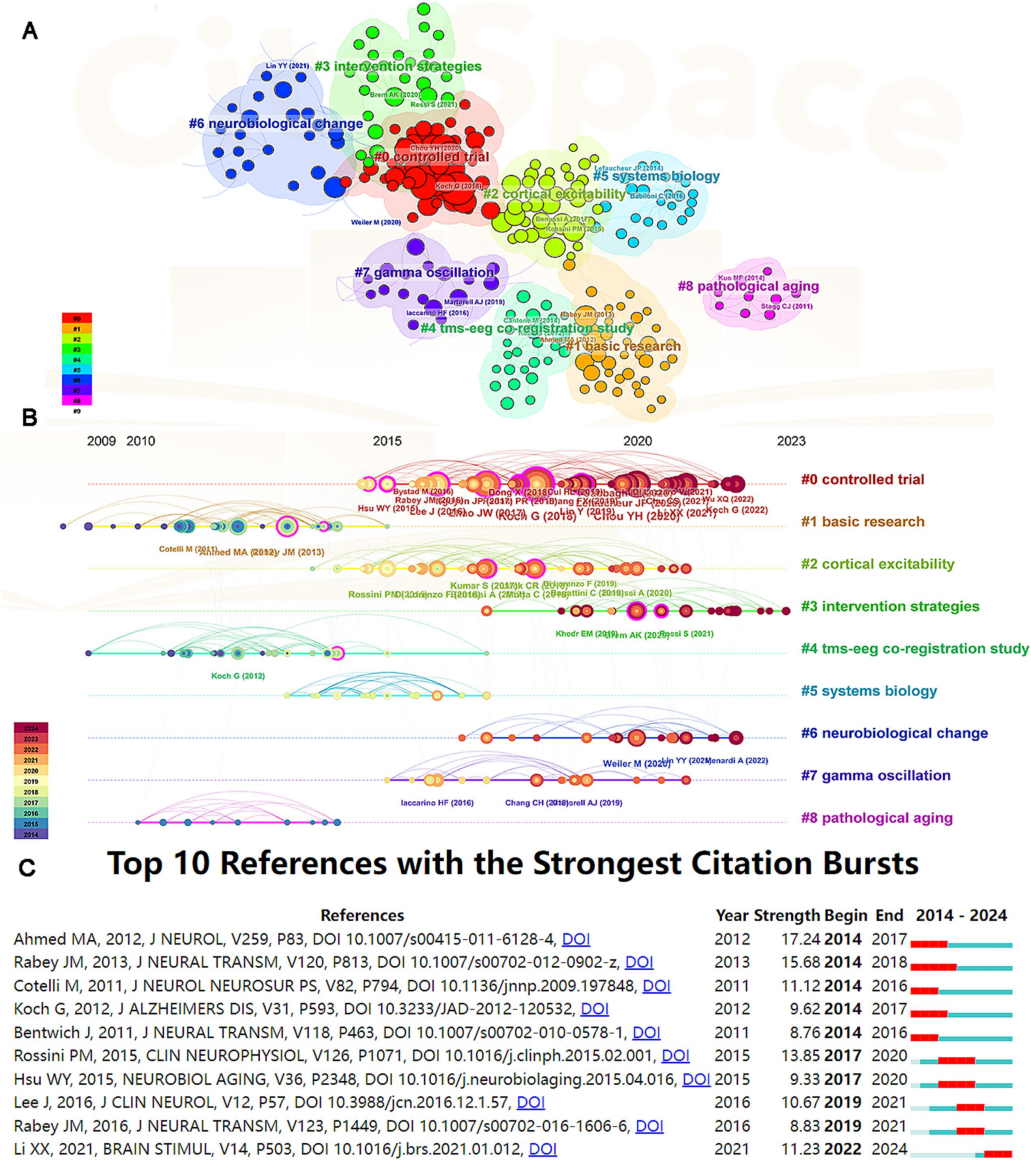
Analysis of references. **(A)** The clusters of co-cited references. **(B)** The timeline view of co-cited references. **(C)** Citation bursts analysis of references.

Table 2 lists the top 10 most frequently cited references in the research on Alzheimer’s disease and TMS. The most cited article is “Transcranial magnetic stimulation of the precuneus enhances memory and neural activity in prodromal Alzheimer’s disease,” authored by Koch et al. published in NEUROIMAGE in 2018, with 81 citations (Koch et al., 2018). Following that, Chou YH et al. published “A systematic review and meta-analysis of rTMS effects on cognitive enhancement in mild cognitive impairment and Alzheimer’s disease” in NEUROBIOL AGING in 2020, which received 71 citations (Chou et al., 2020). Additionally, the paper “Repetitive transcranial magnetic stimulation improves cognitive function of Alzheimer’s disease patients” by Zhao et al. published in ONCOTARGET in 2017, garnered 50 citations (Zhao et al., 2017). Co-citation analysis conducted using CiteSpace categorized the cited references into several clusters, including controlled trial, basic research, cortical excitability, intervention strategies, TMS-EEG co-registration study, systems biology, neurobiological change, gamma oscillation, and pathological aging, totaling nine clusters (Figure 7A). References related to basic research, TMS-EEG co-registration study, and pathological aging were more prevalent prior to 2015. In contrast, after 2015, there has been a concentration of citations related to controlled trial, cortical excitability, intervention strategies, and neurobiological change (Figure 7B). Citation burst analysis of references revealed a total of 82 citations identified, with the top 10 displayed in Figure 7C. The article with the strongest citation burst is “Effects of low versus high frequencies of repetitive transcranial magnetic stimulation on cognitive function and cortical excitability in Alzheimer’s dementia” by Ahmed et al., published in the Journal of Neurology in 2012 (Strength = 17.24) (Ahmed et al., 2012). Furthermore, the paper “Cortical plasticity is correlated with cognitive improvement in Alzheimer’s disease patients after rTMS treatment” by Li et al. published in BRAIN STIMUL in 2021 (Li et al., 2021), is currently still in its citation burst period.

**Table 2:** Top 10 cited references by citations.

Title	Journal	First author	Year	Total citation frequency
Transcranial magnetic stimulation of the precuneus enhances memory and neural activity in prodromal Alzheimer’s disease	NEUROIMAGE	Koch G	2018	81
A systematic review and meta-analysis of rTMS effects on cognitive enhancement in mild cognitive impairment and Alzheimer’s disease	NEUROBIOL AGING	Chou YH	2020	71
Repetitive transcranial magnetic stimulation improves cognitive function of Alzheimer’s disease patients	ONCOTARGET	Zhao JW	2017	50
Evidence-based guidelines on the therapeutic use of repetitive transcranial magnetic stimulation (rTMS): An update (2014–2018)	CLIN NEUROPHYSIOL	Lefaucheur JP	2020	48
Effects of a combined transcranial magnetic stimulation (TMS) and cognitive training intervention in patients with Alzheimer’s disease	ALZHEIMERS DEMENT	Sabbagh M	2020	47
Cortical plasticity is correlated with cognitive improvement in Alzheimer’s disease patients after rTMS treatment	BRAIN STIMUL	Li XX	2021	44
The role of repetitive transcranial magnetic stimulation (rTMS) in the treatment of cognitive impairment in patients with Alzheimer’s disease: A systematic review and meta-analysis	J NEUROL SCI	Lin Y	2019	40
Treatment of Alzheimer’s disease with Repetitive Transcranial Magnetic Stimulation Combined with Cognitive Training: A Prospective, Randomized, Double-Blind, Placebo-Controlled Study	J CLIN NEUROL	Lee J	2016	36
High-frequency repetitive transcranial magnetic stimulation combined with cognitive training improves cognitive function and cortical metabolic ratios in Alzheimer’s disease	J NEURAL TRANSM	Zhang FX	2019	33
Repetitive transcranial magnetic stimulation for cognitive impairment in Alzheimer’s disease: a meta-analysis of randomized controlled trials	J NEUROL	Wang X	2020	33

### 3.7 Analysis of keywords

**Figure 8:**
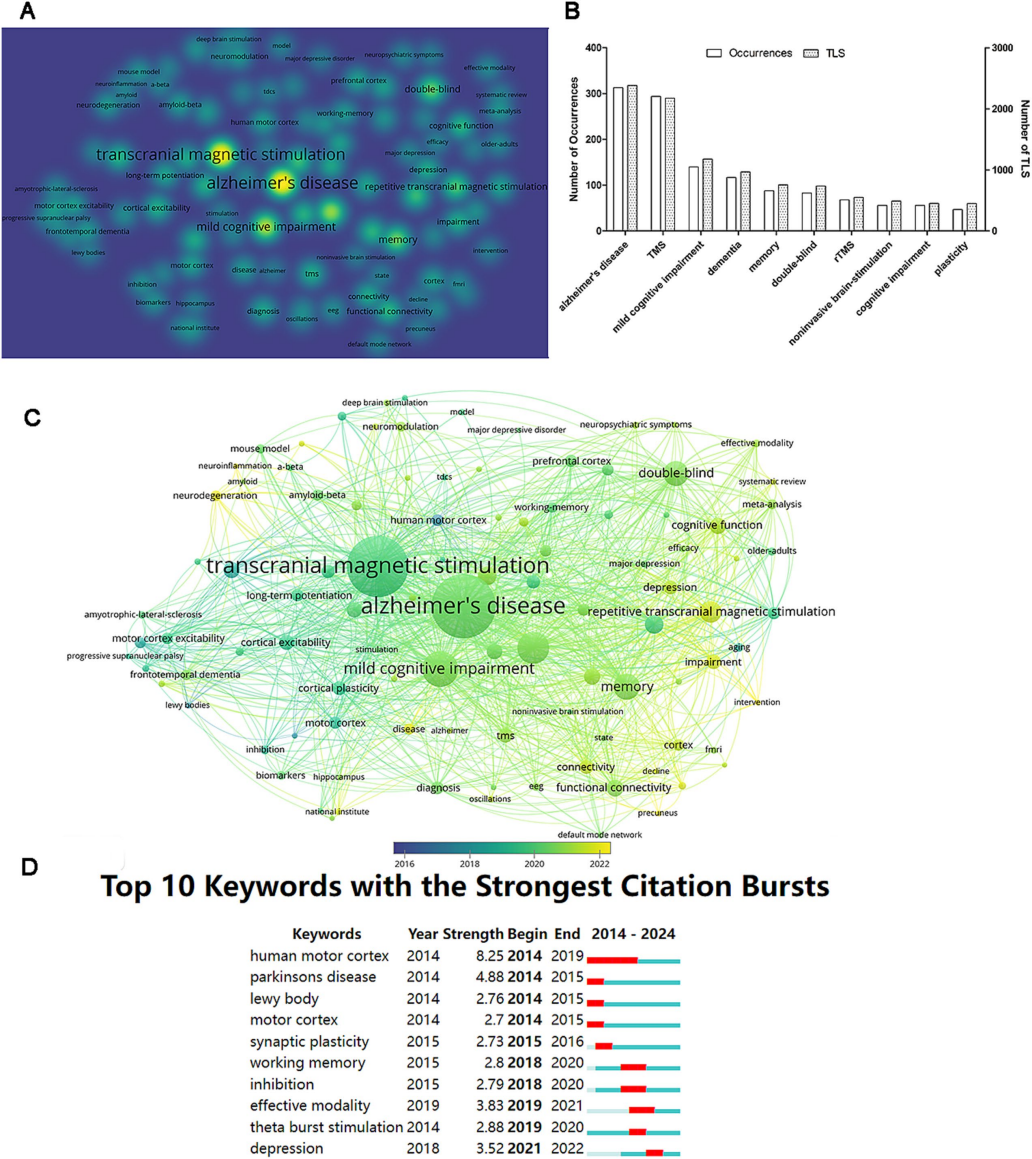
Analysis of keywords. **(A)** The density visualization of keywords based on occurrence numbers. **(B)** The 10 most frequent keywords. **(C)** The overlay visualization of keywords. **(D)** Citation burst analysis of keywords.

The goal of co-occurrence analysis plays a vital role in monitoring the progression of scientific developments by exploring prevalent research directions and fields. Using VOSviewer, we selected and analyzed keywords appearing in at least 10 papers, resulting in the identification of 106 keywords (Figure 8A). The top 10 keywords based on frequency are: Alzheimer’s disease, transcranial magnetic stimulation (TMS), mild cognitive impairment, dementia, memory, double-blind, repetitive transcranial magnetic stimulation (rTMS), noninvasive brain stimulation, cognitive impairment, and plasticity (Figure 8B). The overlay visualization view indicates that keywords such as neurodegeneration, neuroinflammation, and intervention have gained prominence in recent years (Figure 8C). Furthermore, the citation bursts analysis of keywords demonstrates that a total of 13 keywords experienced citation bursts. The top 10 keywords are illustrated in Figure 8D, with human motor cortex exhibiting the strongest citation burst (strength = 8.25) and the longest duration of citation bursts (2014–2019).

### 3.8 Text mining

**Figure 9:**
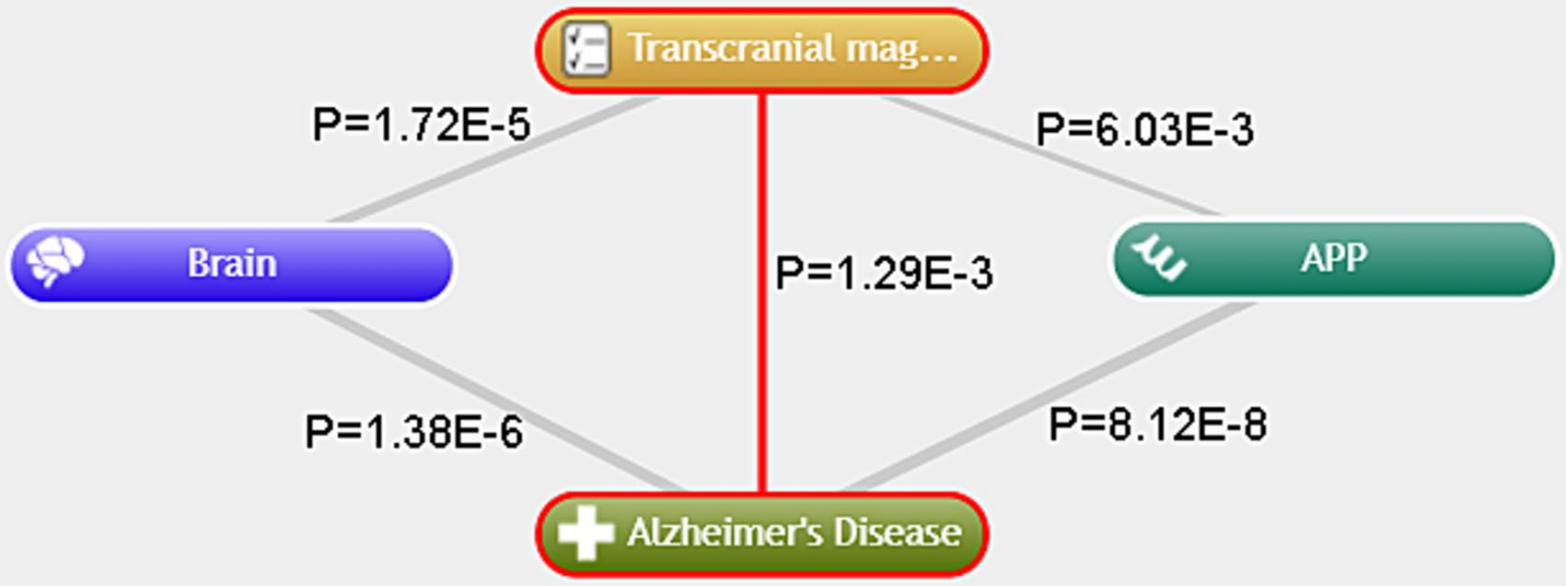
Text mining analysis conducted with CoreMine.

The use of CoreMine for text mining reveals significant associations in the anatomical structure categories, indicating that “brain” shows a notable connection with both “Alzheimer’s disease” and “TMS” (*p* < 0.05). In the gene categories, “Amyloid Precursor Protein (APP)” also demonstrates a significant relationship with both AD and TMS (*p* < 0.05) (Figure 9). This suggests that TMS may potentially serve as a therapeutic approach for AD through the modulation of APP.

## 4 Discussion

The role of TMS in AD has sparked significant interest among researchers. This area of study continues to grow each year, especially following 2020, as shown in Figure 2 of our article. Given the surge in published articles and the ongoing trend, it is essential for researchers to grasp the research trends, hotspots, and existing challenges within this field. Thus, conducting a bibliometric analysis of the correlation between TMS and AD is crucial. Such analysis can provide researchers with objective data from past literature, offering a clear and comprehensive view of research trends, hotspots, and challenges. This insight will enable future studies to be more focused, ultimately aiding researchers in determining the effects of TMS on AD and exploring its potential mechanisms.

China has published the largest number of articles in this field, given most academic articles published internationally having Chinese scholars as their leading and corresponding authors. In the near future, scholars in other countries all over the world could consider increasing the citation rate and the level of international collaboration. As expected, the number of articles published, citation counts, and levels of international cooperation in some underdeveloped countries such as Italy rank much lower than China as the top country, indicating the need to learn from China and strengthen research collaboration with China in this field, which could improve the research capabilities among the collaborating countries. The number of articles published, citation counts, and levels of international cooperation in the US and Italy rank among the top three, indicating their research strengths in this field. Factors such as severe aging, advanced healthcare levels, and high rates of research collaboration likely contribute to the significant influence these two countries exert in this area (Nichols et al., 2022). Importantly, this leading impact continues as evidenced by citation bursts analysis, with institutions such as the University of Ferrara and Massachusetts General Hospital, along with Italian author Martina Assogna, currently experiencing citation surges.

A comprehensive statistical examination of scholarly publication volumes reveals a striking trend: the foremost 10 journals consistently secure placements in the Q2 or higher within the esteemed Journal Citation Reports (JCR) rankings. This trend underscores the pronounced interest that research pertaining to AD and TMS has attracted from prestigious academic publications. The dissemination of research papers in these highly regarded journals not only elevates public awareness but also stimulates increased scholarly interest in the practical applications of TMS for AD treatment. Furthermore, the implementation of bibliometric coupling analysis encompassing these journals holds significant promise for researchers. By leveraging such analytical methods, scholars can gain insights into contemporary publishing trends and preferences regarding articles focused on AD and TMS. This strategic approach enables researchers to make informed choices regarding suitable journals for manuscript submissions. Additionally, it facilitates effective monitoring of advancements within the research landscape, ensuring that academics remain connected to the evolving discourse surrounding innovative treatment modalities for AD.

A thorough analysis of references and targeted keywords sheds light on the primary research focal points and trending topics within the realm of TMS and AD. The findings reveal that literature garnering significant citations predominantly centers on clinical trials, as well as comprehensive meta-analyses concerning TMS applications in AD contexts. Notably, keywords like “double-blind” and “intervention” frequently surface, indicating a robust interest in the methodological rigor of clinical trials associated with TMS. This pattern highlights an overall concern regarding the therapeutic efficacy of TMS in treating AD. Recent studies employing functional magnetic resonance imaging (fMRI) provide compelling evidence that TMS can indeed generate neuroplastic changes in both the directly stimulated cortical regions and the areas of the brain that are interconnected. Nonetheless, it is important to note that these neuroplastic effects tend to be transient in nature, raising questions about the long-term benefits of TMS in clinical practice. As such, the effectiveness of TMS as a treatment modality for alleviating cognitive decline associated with AD remains an area of ongoing inquiry and debate (D'Amelio and Di Lazzaro, 2023). In light of these considerations, research into the influence of TMS on cognitive function in AD continues to garner sustained and elevated attention from the scientific community.

Amyloid plaques represent a significant pathological hallmark of AD, a neurodegenerative disorder that devastates cognitive functions (Bloom, 2014). The APP plays a pivotal role as a precursor for the generation of beta-amyloid peptides. Dysregulation within the multifaceted pathways associated with APP processing can precipitate the onset and progression of AD (O'Brien and Wong, 2011). Text mining techniques have revealed a noteworthy correlation between APP and both AD and TMS. This correlation implies that TMS may offer a therapeutic avenue for AD by modulating the activity of APP. Recent experimental investigations have demonstrated that following TMS intervention, there is a notable reduction in neuronal damage within the hippocampal formation of murine models. Concurrently, the expression levels of APP and phosphorylated Thr231 exhibit a decline, while the spontaneous firing rates of neurons in select brain regions show an uptick. In APP/PS1 transgenic mouse models (Huang et al., 2023), TMS has been shown to influence mitochondrial respiration and operational capacity through the mediation of ISCA1, which is integral to iron–sulfur cluster assembly. This modulation alleviates cognitive impairments and mitigates the pathological features associated with Alzheimer’s disease. Moreover, TMS appears to attenuate neuroinflammatory processes, reduce neuronal apoptosis, and prevent synaptic degradation in APP/PS1 mouse models. This effect is attributed to the inhibition of amyloid-beta (Aβ) peptide production, alongside the enhancement of Aβ degradation pathways (Zhu et al., 2024). Nonetheless, the exact mechanisms through which TMS yields long-term benefits in ameliorating APP/PS1 pathology, and its specific implications for APP expression within the human brain, remain inadequately evidenced. This lack of clarity presents significant scientific inquiries that demand further exploration and rigorous investigation in subsequent research endeavors.

## 5 Limitations

This study exclusively retrieved and analyzed data on AD and TMS from the Web of Science database, without incorporating information from other databases such as PubMed, Google Scholar, or Scopus. Additionally, the inclusion criteria were limited to literature published in English, thereby excluding studies available in other languages, including those from Chinese databases like CNKI. As a result, some potentially relevant studies may not have been captured in this analysis. Furthermore, TMS consists of various stimulation methods, such as Single Pulse, Paired Pulse, and Repetitive. Different parameters for TMS might yield varying effects on AD. Thus, when sufficient literature is available, it is crucial to refine the different TMS treatment parameters for bibliometric analysis.

## 6 Conclusion

In conclusion, this manuscript implements advanced bibliometric techniques to perform a thorough and objective quantitative examination of the published literature in the specialized domains of AD and TMS. This approach not only elucidates the contemporary landscape of research activities in these fields but also pinpoints significant research hotspots that have garnered attention among scholars. Additionally, it systematically addresses the prevailing hurdles that researchers currently encounter, thereby offering a foundational reference for guiding future investigations and encouraging further exploration in these critical areas of study. The insights derived from this analysis hold potential implications for the direction of forthcoming research initiatives and could significantly contribute to the advancement of therapeutic strategies for AD through innovative applications of TMS.

## Data Availability

The original contributions presented in the study are included in the article/supplementary material, further inquiries can be directed to the corresponding authors.

## Footnotes

^1^ https://bibliometric.com/

^2^ https://coremine.com/medical/#search

## References

[ref1] Ahmed M. A. Darwish E. S. Khedr E. M. El Serogy Y. M. Ali A. M. (2012). Effects of low versus high frequencies of repetitive transcranial magnetic stimulation on cognitive function and cortical excitability in Alzheimer's dementia. J. Neurol. 259, 83–92. doi: 10.1007/s00415-011-6128-4, PMID: 21671144

[ref2] Bloom G. S. (2014). Amyloid-β and tau: the trigger and bullet in Alzheimer disease pathogenesis. JAMA Neurol. 71, 505–508. doi: 10.1001/jamaneurol.2013.5847, PMID: 24493463 PMC12908160

[ref3] Chang C. H. Lane H. Y. Lin C. H. (2018). Brain stimulation in Alzheimer's disease. Front. Psych. 9:201. doi: 10.3389/fpsyt.2018.00201, PMID: 29910746 PMC5992378

[ref4] Chou Y. H. Sundman M. Ton That V. Green J. Trapani C. (2022). Cortical excitability and plasticity in Alzheimer's disease and mild cognitive impairment: a systematic review and meta-analysis of transcranial magnetic stimulation studies. Ageing Res. Rev. 79:101660. doi: 10.1016/j.arr.2022.101660, PMID: 35680080 PMC9707650

[ref5] Chou Y. H. Ton That V. Sundman M. (2020). A systematic review and meta-analysis of rTMS effects on cognitive enhancement in mild cognitive impairment and Alzheimer's disease. Neurobiol. Aging 86, 1–10. doi: 10.1016/j.neurobiolaging.2019.08.020, PMID: 31783330 PMC6995441

[ref6] D'Amelio M. Di Lazzaro V. (2023). Can transcranial magnetic stimulation rescue dopaminergic signalling in Alzheimer's disease? Brain 146, e43–e45. doi: 10.1093/brain/awad019, PMID: 36729723 PMC10232239

[ref8] Graff-Radford J. Yong K. X. X. Apostolova L. G. Bouwman F. H. Carrillo M. Dickerson B. C.. (2021). New insights into atypical Alzheimer's disease in the era of biomarkers. Lancet Neurol. 20, 222–234. doi: 10.1016/s1474-4422(20)30440-3, PMID: 33609479 PMC8056394

[ref9] Hallett M. (2007). Transcranial magnetic stimulation: a primer. Neuron 55, 187–199. doi: 10.1016/j.neuron.2007.06.026, PMID: 17640522

[ref10] Huang H. Zhu Y. Liao L. Gao S. Tao Y. Fang X.. (2023). The long-term effects of intermittent theta burst stimulation on Alzheimer's disease-type pathologies in APP/PS1 mice. Brain Res. Bull. 202:110735. doi: 10.1016/j.brainresbull.2023.110735, PMID: 37586425

[ref11] Khan S. Barve K. H. Kumar M. S. (2020). Recent advancements in pathogenesis, diagnostics and treatment of Alzheimer's disease. Curr. Neuropharmacol. 18, 1106–1125. doi: 10.2174/1570159x18666200528142429, PMID: 32484110 PMC7709159

[ref12] Koch G. Bonnì S. Pellicciari M. C. Casula E. P. Mancini M. Esposito R.. (2018). Transcranial magnetic stimulation of the precuneus enhances memory and neural activity in prodromal Alzheimer's disease. Neuroimage 169, 302–311. doi: 10.1016/j.neuroimage.2017.12.048, PMID: 29277405

[ref13] Li X. Qi G. Yu C. Lian G. Zheng H. Wu S.. (2021). Cortical plasticity is correlated with cognitive improvement in Alzheimer's disease patients after rTMS treatment. Brain Stimul. 14, 503–510. doi: 10.1016/j.brs.2021.01.012, PMID: 33581283

[ref14] Matt E. A.-O. Dörl G. Beisteiner R. (2022). Transcranial pulse stimulation (TPS) improves depression in AD patients on state-of-the-art treatment. Alzheimers Dement 8:e12245. doi: 10.1002/trc2.12245 PMC8829892 35169611

[ref15] Mullard A. (2021). Failure of first anti-tau antibody in Alzheimer disease highlights risks of history repeating. Nat. Rev. Drug Discov. 20, 3–5. doi: 10.1038/d41573-020-00217-7, PMID: 33303932

[ref7] Nichols E. Steinmetz J. D. Vollset S. E. Fukutaki K. Chalek J. Abd-Allah F.. (2022). Estimation of the global prevalence of dementia in 2019 and forecasted prevalence in 2050: an analysis for the global burden of disease study 2019. Lancet Public Health 7, e105–e125. doi: 10.1016/s2468-2667(21)00249-8, PMID: 34998485 PMC8810394

[ref16] O'Brien R. J. Wong P. C. (2011). Amyloid precursor protein processing and Alzheimer's disease. Annu. Rev. Neurosci. 34, 185–204. doi: 10.1146/annurev-neuro-061010-113613, PMID: 21456963 PMC3174086

[ref17] Scheltens P. de B. Kivipelto M. Holstege H. Chételat G. Teunissen C. E.. (2021). Alzheimer's disease. Lancet 397, 1577–1590. doi: 10.1016/s0140-6736(20)32205-4, PMID: 33667416 PMC8354300

[ref18] Tahami Monfared A. A. Byrnes M. J. White L. A. Zhang Q. (2022). The humanistic and economic burden of Alzheimer's disease. Neurol Ther 11, 525–551. doi: 10.1007/s40120-022-00335-x, PMID: 35192176 PMC9095804

[ref19] Vaishnavi S. (2023). Transcranial magnetic stimulation for developmental neuropsychiatric disorders with inflammation. Dev. Neurosci. 45, 342–348. doi: 10.1159/000535103, PMID: 37944502 PMC10664335

[ref20] Zhao J. Li Z. Cong Y. Zhang J. Tan M. Zhang H.. (2017). Repetitive transcranial magnetic stimulation improves cognitive function of Alzheimer's disease patients. Oncotarget 8, 33864–33871. doi: 10.18632/oncotarget.13060, PMID: 27823981 PMC5464918

[ref21] Zhu Y. Huang H. Chen Z. Tao Y. Liao L. Y. Gao S. H.. (2024). Intermittent Theta burst stimulation attenuates cognitive deficits and Alzheimer's disease-type pathologies via ISCA1-mediated mitochondrial modulation in APP/PS1 mice. Neurosci. Bull. 40, 182–200. doi: 10.1007/s12264-023-01098-7, PMID: 37578635 PMC10838862

